# RNA Directed Modulation of Phenotypic Plasticity in Human Cells

**DOI:** 10.1371/journal.pone.0152424

**Published:** 2016-04-15

**Authors:** Laura Trakman, Chris Hewson, Jon Burdach, Kevin V. Morris

**Affiliations:** 1 The University of New South Wales, Biotechnology and Biomedical Sciences, Sydney NSW 2052, Australia; 2 City of Hope – Beckman Research Institute, Center for Gene Therapy, Duarte, California, 91010, United States of America; George Mason University, UNITED STATES

## Abstract

Natural selective processes have been known to drive phenotypic plasticity, which is the emergence of different phenotypes from one genome following environmental stimulation. Long non-coding RNAs (lncRNAs) have been observed to modulate transcriptional and epigenetic states of genes in human cells. We surmised that lncRNAs are governors of phenotypic plasticity and drive natural selective processes through epigenetic modulation of gene expression. Using heat shocked human cells as a model we find several differentially expressed transcripts with the top candidates being lncRNAs derived from retro-elements. One particular retro-element derived transcripts, Retro-EIF2S2, was found to be abundantly over-expressed in heat shocked cells. Over-expression of Retro-EIF2S2 significantly enhanced cell viability and modulated a predisposition for an adherent cellular phenotype upon heat shock. Mechanistically, we find that this retro-element derived transcript interacts directly with a network of proteins including 40S ribosomal protein S30 (FAU), Eukaryotic translation initiation factor 5A (EIF5A), and Ubiquitin-60S ribosomal protein L40 (UBA52) to affect protein modulated cell adhesion pathways. We find one motif in Retro-EIF2S2 that exhibits binding to FAU and modulates phenotypic cell transitions from adherent to suspension states. The observations presented here suggest that retroviral derived transcripts actively modulate phenotypic plasticity in human cells in response to environmental selective pressures and suggest that natural selection may play out through the action of retro-elements in human cells.

## Introduction

Phenotypic plasticity is the emergence of different phenotypes from one genome following environmental stimulation and has been argued to be the result of natural selection. Indeed, plasticity is best demonstrated by heat shocking cells, where entirely different transcriptional profiles are expressed including those of centromeric repeat regions of the genome [1], heat shock 70 genes [2], and SINE elements [3]. Collectively these observations suggest that transcription plays a significant role in the mechanism of phenotypic plasticity.

Non-coding RNAs have been found to function as modulators of epigenetic states in human cells [4]. Long non-coding RNAs (lncRNAs) are transcripts greater than 200 nucleotides in length that share similarities with messenger RNA. LncRNAs are pervasive throughout the transcriptome of eukaryotes [5] and have been shown to be involved in the transcriptional and epigenetic regulation of gene expression [6–9]. Several observations have now been reported on particular subsets of lncRNAs that overlap the 5’ regions of protein-coding genes [10], with some found to modulate the transcriptional and epigenetic states of their homology containing target gene [6,11–13]. Collectively, these observations suggest that non-coding RNAs may play a role in cellular adaptation to environmental cues through governing phenotypic plasticity.

## Materials and Methods

Cell Culture- HEK293 cells were cultured in DMEM which was supplemented with 1% Penicillin-Streptomycin-Glutamine solution and 10% FBS. The cells were incubated at 37°C and 5% CO_2_.

Heat Shock of HEK293 cells- During cell passaging, an aliquot of cells was taken and placed in a 15ml falcon tube. The falcon tube was placed in a water bath at 42, 45, 48 or 50°C for 30 minutes depending on the parameters of the outlined experiment.

Deep sequencing analysis of Heat shock treated 293HEK cells- Quality control was performed using FastQC v0.10.1 available from http://www.bioinformatics.babraham.ac.uk/projects/fastqc/. Reads were quality filtered, trimmed and adapter sequences were removed using Trimmomatic v0.3.0 [14]. Reads were aligned to the hg19 Homo sapiens genome using tophat2 using the default settings except for the following (-M -microexon-search -n 3—genome-read-mismatches 3—read-mismatches 3 —b2-sensitive) [15]. Transcripts were assembled using Cufflinks v2.0.2 and the hg19 annotations were included [16]. Transcripts across all replicates were merged using cuffmerge and differential expression analysis was performed pair-wise on the groups using cuffdiff (S1 Table). The accession for these data is GSE79680GEO and can be found at https://www.ncbi.nlm.nih.gov/geo/query/acc.cgi?acc=GSE79680.

siRNA suppression heat shock studies- SiRNA pools targeted to UBA52, FAU and EIF5A (Dharmacon, M-013149-01-0005, M-011794-01-0005, L-015739-00-0005) and the control scrambled siRNA (Dharmacon) were transfected into 293HEK cells and 24hrs later the cells were heatshocked (45°C, 30 minutes) and the viability of suspension vs. adherent cells was determined 48hrs later (Muse Cell analyser, Merck Millipore). Suppression of UBA52, FAU and EIF5A was determined by qPCR with the various gene specific primers (S2 Table) relative to control cells, scrambled siRNA transfected, cells alone and/or Mock treated.

Measuring cell viability of adherent and suspension cells using the Muse Cell Analyser- The viability of suspension cells was determined by centrifugation (700 x g for 5 minutes) of culture supernatants. The pelleted cells were resuspended in 200μl of PBS to achieve the desired cell concentration for the Millipore Muse Cell Analyser. 50μl of this cell suspension was mixed with 450μl of Muse Cell Viability reagent and incubated for 5 minutes before being run on the Muse Cell Analyse (as described in manufactures recommendations, Muse Cell analyser, Merck Millipore). The viability of adherent cells was measure by resuspending the adherent cells (500μl of DMEM) and 50μl of these suspended cells was taken and mixed with 450μl of Muse Cell Viability reagent and incubated for 5 minutes at room temperature. The cells were then run on the Muse Cell Analyser.

Generation of Retro-EIF2S2 and Retro-Cox6A RNA expressing clones- The parental pcDNA3.1 plasmid was used and the respective lncRNA sequences of either Retro-Cox6A or Retro-EIF2S2, or truncated forms of Retro-EIF2S2 (S3 Table) were synthesized and generated commercially (Genewiz, Inc La Jolla, CA, USA). Midi prepped (QIAGEN plasmid Midi Kit) clones were transfected along with the pcDNA3.1-GFP control [17] into target cells using lipofectamine 2000 (Life Technologies, Carlsbad, CA, USA).

Generating Biotin Labeled RNA Transcripts- Biotin labeled RNA transcripts of Retro-EFI2S2 and GFP were generated by adding the following components into an 8 well PCR strip: (2.5μg of DNA template, 2μl T7 Enzyme mix (Sigma), 1ul rATP, rGTP, rCTP, rUTP (Promega), 1μl Biotin-14-CTP (Life Technologies), 2.5μl 1M DTT (Sigma) and 1μl RNase Inhibitor (Life Technologies)(as described in [17]). The reaction volume was made up to 50μl with RNase free water (QIAGEN). The T7 transcription was carried out at 37°C for 6 hours, which was then inactivated by adding 2μl of 0.5M EDTA. In order to digest the DNA template, DNase digestion was performed on the sample (Ambion Turbo DNA-free, Life Technologies). This was followed by an ethanol precipitation to purify and clean up the Biotin RNAs. The yield and purity was determined using a Nanodrop.

RNA Extraction, cDNA conversion and qPCR- RNA was extracted from the differentially treated cultures, depending on the particular experiment, using the QIAGEN RNeasy Mini Kit and the yield and purity was measured using a Nanodrop. The RNA was digested using the Ambion Turbo DNA-free kit. The RNA was converted to cDNA using M-MLV Reverse Transcriptase (Sigma) and random hexamers (IDT) and qPCR was performed using KAPA Fast SYBRGreen (Kappa Biosystems) with corresponding primers (S3 Table) on the ViiA 7 Real Time PCR system. The qPCR was carried out using the following parameters: 95°C for 3 min followed by 95°C for 3 s and 60°C for 30 s for 40 cycles.

Biotin antisense oligo IP and Liquid chromatography (LC)- A CHIP was carried out 30hrs post-transfection on cultures treated with Retro-EIF2S22 or GFP biotin linked RNAs. The resulting elutes were subjected to Mass Spec analysis. Mass spec was carried out on the resulting IP elutes (as described in [18])(Fee for service Bioanalytical Mass Spectrometry Facility, Mark Wainwright Analytical Centre, UNSW, Sydney Australia). The top-candidate proteins between both Retro-EIF2S2 and not found in the GFP controls were annotated and are shown.

RIP for the Retro-EIF2S2 association with FAU and EIF5A- Prior to the RIP, cells were heatshocked at 48°C for 30 minutes followed by incubation at 37°C, 5% CO_2_ for 72 hours. Cells were then cross-linked using 1% Formaldehyde and quenched using glycine to a concentration of 0.125M. The samples were lysed using cell lysis buffer (50mM HEPES, 140mM NaCL, 1%v/v Triton X-100, 0.1% Sodium Deoxycholate), sonicated, and the cell debris was removed via centrifugation. An input was taken and the remaining sample was split equally into 2 different tubes. The antibodies to pull down the desired protein (Anti-FAU (ab63065) and Anti-eIF5a (ab32443)) were added to one tube while an IGG antibody (Sigma) was added to the other. This was followed by an overnight incubation at 4°C. After the incubation, the antibodies were captured using Protein G beads (Life technologies) and then washed with Low Salt Wash Buffer (0.1% SDS, 1% Triton X-100, 2mM EDTA, 20mM Tris-HCl, 150 mM NaCl), High Salt Wash Buffer (0.1% SDS, 1% Triton X-100, 2mM EDTA, 1-mM Tris-HCl, 0.5M NaCl), Lithium Chloride Wash Buffer (0.25M LiCl, 1% NP-40, 1% Sodium Deoxycholate, 1mM EDTA, 10mM Tris-HCl) followed by a final wash with Low Salt Wash buffer. After the final wash, the antibodies were eluted using an elution buffer (10mM Tris-HCl, 1mM EDTA, 2.0M NaCl). The samples were reverse-crosslinked overnight followed by Proteinase K and DNase treatment. The RNA was extracted using TRIzol (Life Technolgoies) and cleaned up using the QIAGEN RNeasy Mini Kit. The RNA was converted to cDNA and then analysed on the Viia7 Real Time PCR system with Retro-EIF2S2 specific primers (S2 Table).

## Results

**Fig 1:**
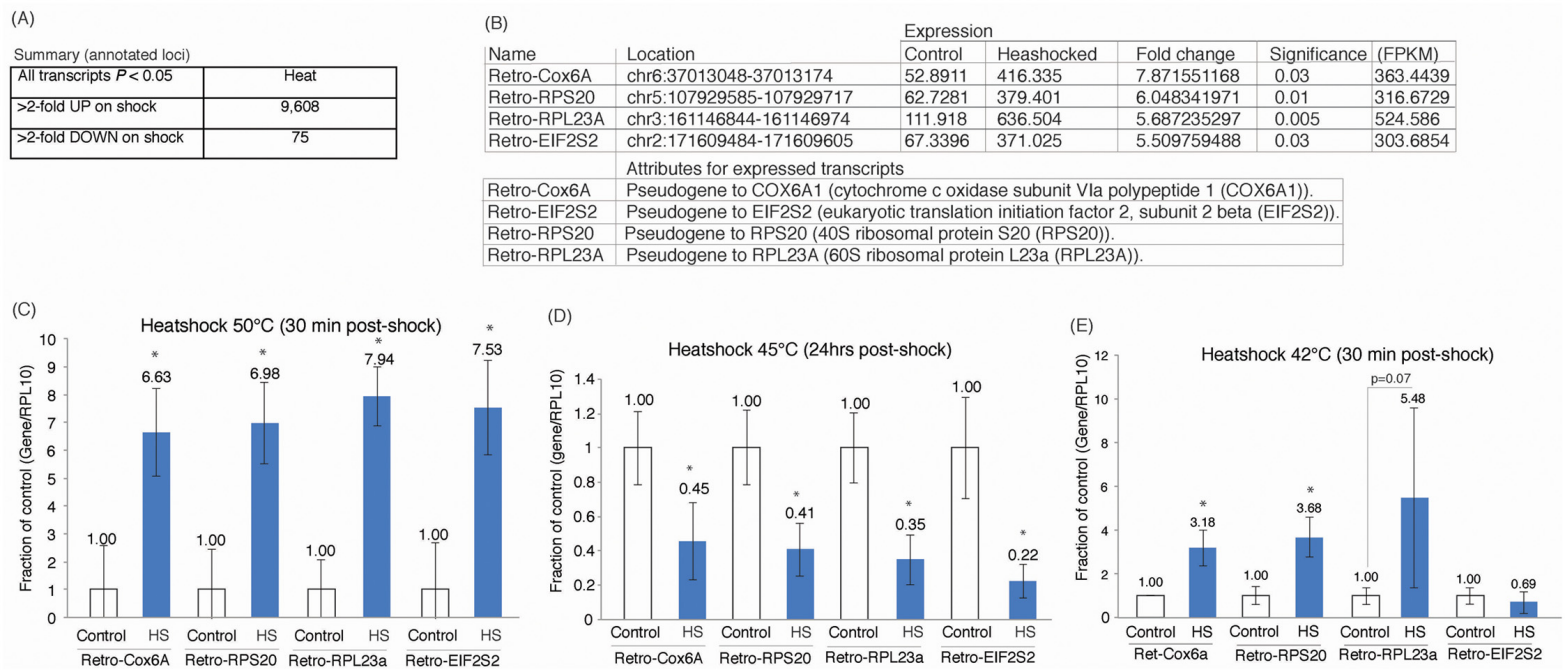
The effects of heat shock on gene expression and the top candidate lncRNAs differentially modulated. (**A**) The summary of differentially expressed genes at 30 minutes post heat shock (45°C) in HEK293 cells was determined by transcriptome deep sequencing. (**B**) The top candidate heat-shock induced transcripts containing retroviral derived retrogenes that were experimentally validated further. (**C**) The top-candidate retrogenes are also increased in expression 30 minutes following heat shock at 50°C. (**D**) The top-candidate retrogene expression is significantly reduced 24hrs post-heat shock. (**E**) Retro-Cox6A, RPS20 and RPL32a are increased in expression while Retro-EIF2S2 is not affected by heat shock at 42°C. For C-E the averages of triplicate treated cultures are shown with the standard deviations and * indicates significant differences (p<0.05) as determined by a paired two-sided T-test.

To determine the role of non-coding RNAs in the modulation of cellular plasticity, we heat shocked HEK293 cells and determined those transcripts that become differentially expressed by 30 minutes post-heatshock (Fig 1A and S1 Fig). Thirty minutes following heat shock there appeared to be an immediate and significant increase in the expression of 9,608 genes, including a large subset found to have pseudogene and retro-element transcript counterparts (Fig 1A and 1B, S3 Table). Interestingly, a subset of retro-elements to Cox6a, RPS20, RPL23A and EIF2S2, well-known protein-coding genes involved in cellular stress-responses, were significantly expressed upon heat shock (Fig 1B and 1C), but this effect was lost after 24hrs post-heat shock, suggesting possibly that the observed expression of Cox6a, RPS20, RPL23A and EIF2S2 transcripts 30 minutes post-heat shock are a transcriptional response to heat stress (Fig 1D). Notably, only the retro-elements Cox6a, RPS20 and RPL23 were expressed when cultures were heat shocked at 42°C (Fig 1E). Two of these retro-elements emanated from gene deserts, several KB away from neighboring protein-coding or non-coding transcripts (S2 and S3 Figs). Both of these non-coding genes, Retro-Cox6A and Retro-EIF2S2 (S2 and S3 Figs respectively), had 5 matching pseudogene/retro-element counterparts inserted throughout the genome (S4 Table), and their relative increased expression upon heat shock was ~6–7 fold (Fig 1), suggesting that these transcripts may play an endogenous role in cellular responses to heat shock.

**Fig 2:**
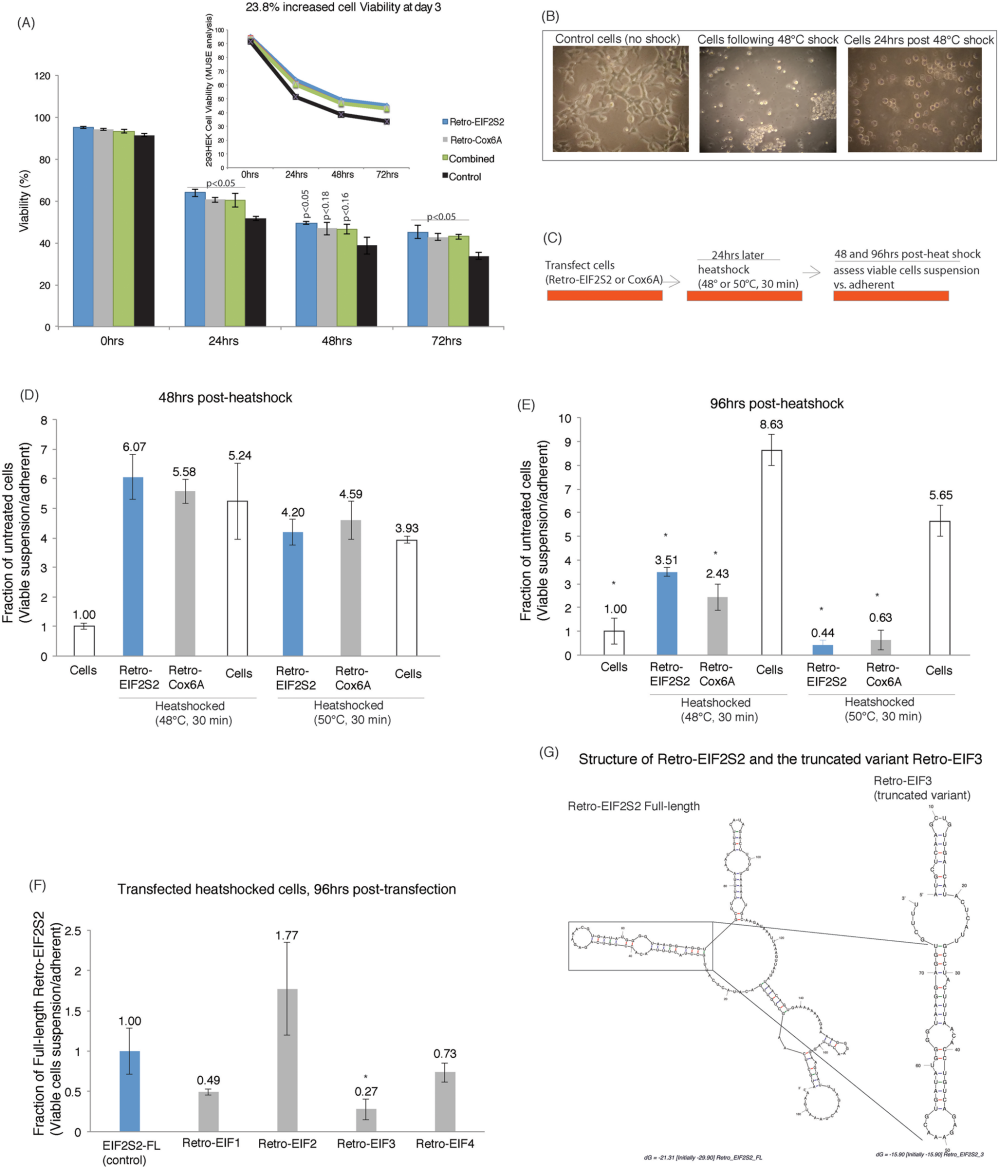
The effects of Retrogene RNA expression on phenotypic plasticity in heat shocked HEK293 cells. (**A**) Differentially transfected HEK293 cells were characterized for the effects of lncRNA expression on cell viability at various times post-transfection. The averages of triplicate treated cells are shown with the standard error of the mean and p values shown from a paired two-sided T-test. (**B**) An image of control and heat shocked adherent and suspension cells immediately after and 24hrs post-heat shock. (**C**) The experimental plan for determining the effects of overexpression of Retro-EIF2S2 and Retro-Cox6A on cellular states during heat shock. (**D-E**) The over-expression of Retro-EIF2S2 and Retro-Cox6A predisposes heat shocked cells at 96hrs post-heat shock towards an adherent state relative to untransfected controls at both 48°C and 50°C. (**E**) The effects of truncated variants of Retro-EIF2S2 to modulate suspension cells relative to the Retro-EIF2S2 in heat shocked cells. (**F**) The truncated variant EIF3 and a focus on the conserved hairpin stem-loop structure relative to full-length Retro-EIF2S2. Transfected and heat shock treated cultures were assessed 96hrs post-head shock (45°C for 30 min). For (A and D-F) the averages of triplicate treated cells are shown with the standard error of the mean and significance p<0.10 shown (*).

To test this notion further and determine to what extent a subset of the candidate transcripts (S2 Table) can functionally affect cell viability we generated and over-expressed clones for both Retro-Cox6A (~127bp) and Retro-EIF2S2 (~185bp)(S4 and S5 Tables). When these transcripts were over expressed a clear ~23% increase in cell viability was observed relative to control-transfected cultures (Fig 2A, S4 and S5 Figs). These observations suggest that Retro-Cox6A Retro-Cox6A and Retro-EIF2S2 provide a function in driving increased cell survival, viability, and possibly plasticity. Following heat-shocked cells over 96 hours resulted in two populations of distinct cellular phenotypes, adherent and suspension. The suspension cellular phenotype was different than those phenotypes observed immediately following heat shock (Fig 2B). To determine to what extent these retro-elements are involved in controlling the heat shock induced populations of suspension cells, we transfected cultures with Retro-Cox6A and Retro-EIF2S2 or the GFP control and heat shocked at both 48°C and 50°C. The cultures were followed for suspension relative to adherent cells over 96hrs (Fig 2C). A predominant and significant increase in the population of suspension cells was observed at 48 hours post-heat shock (Fig 2D). Interestingly, those cultures treated with Retro-Cox6A and Retro-EIF2S2 largely restored an adherent phenotype at 96 hours following heat shock while the control heat-shocked cells did not (Fig 2E), suggesting that these transcripts somehow facilitate the cell transitioning to an adherent state following heat shock.

The full-length Retro-EIF2S2, which demonstrated robust reconstitution of cell adherence following heat shock (Fig 2E), was selected and mechanistically validated in greater detail. To determine what aspect of Retro-EIF2S2 was required for reconstitution of adherent cells following heat shock several truncated variants of Retro-EIF2S2 were generated (S5 Table) and tested for their respective ability to reconstitute adherent phenotypes following heat shock. One truncated variant, Retro-EIF3 was able to reconstitute adherent cellular phenotypes following heat shock more efficiently than the full-length Retro-EIF2S 2 (Fig 2F). The truncated Retro-EIF3 contained a common stem and loop domain that was also found in full-length Retro-EIF2S2 as determined by M-Fold analysis (Fig 2G), suggesting that this domain may be involved in modulating the observed cellular phenotype following heat shock.

**Fig 3:**
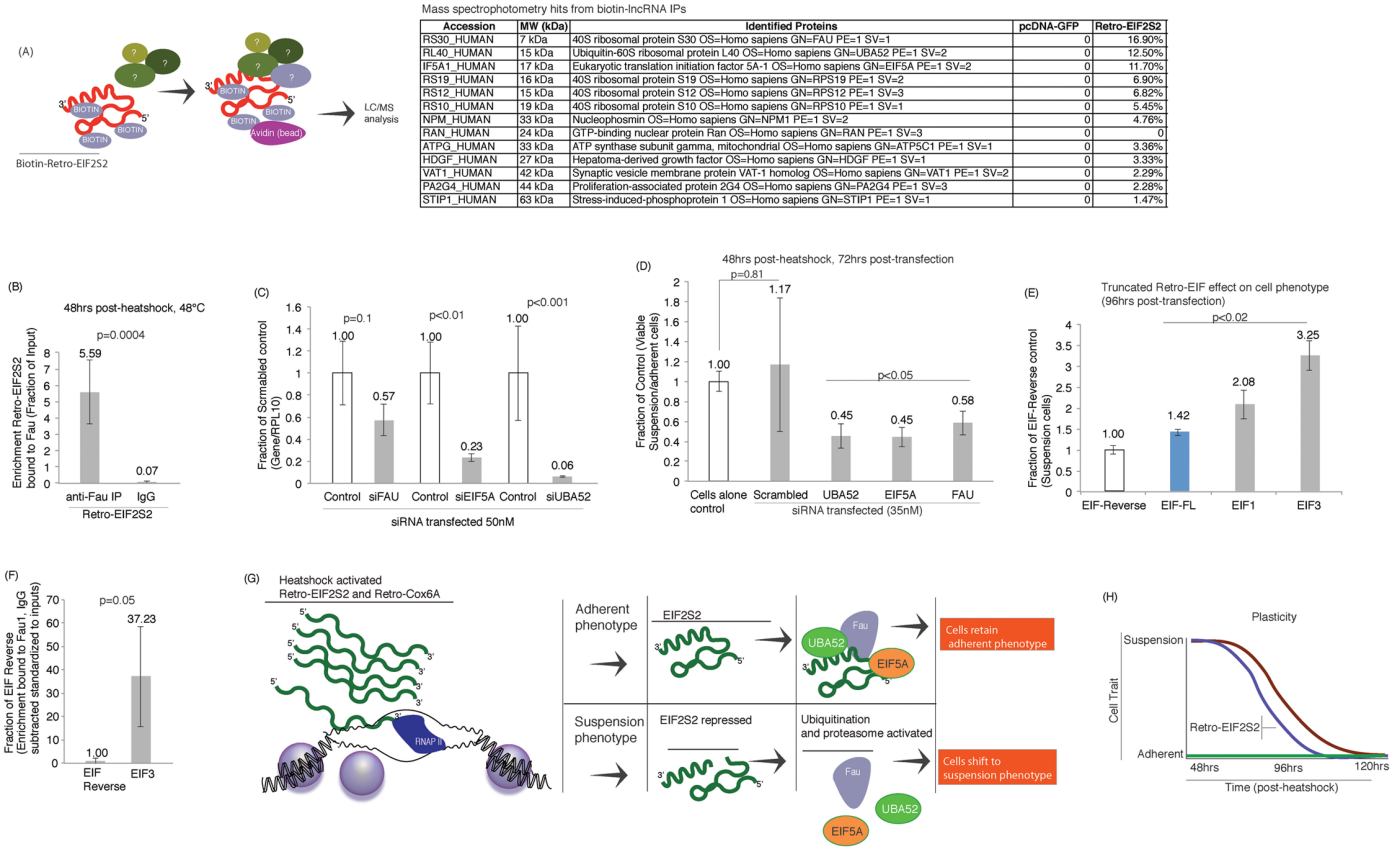
Mechanism of Retro-EIF2S2 modulation of adherent cellular phenotypes. (**A**) Characterization of Retro-EIF2S2 associated proteins. Biotin-labeled Retro-EIF2S2 was immunoprecipated and bound proteins determined relative GFP-Biotin controls. Triplicate transfected cultures were pooled and IP-Mass Spec performed. The top hits are shown with their % coverage of the protein. (**B**) Full-length Retro-EIF2S2 immunoprecipitates with FAU following heat shock. (**C-D**) siRNA repression of (**C**) FAU, EIF5A and UBA (35-50nM siRNA, Dharmacon siRNAs) followed by heat shock results in (**D**) a reduction in viable suspension relative to adherent cell numbers 72hrs post-transfection. (**E**) The over-expression of full-length Retro-EIF1 and truncated EIF3 significantly increase the number of viable suspension cells relative to the control (reverse Retro-EIF2S2). (**F**) The truncated EIF3 binds FAU as determined by RIP relative to the EIF reverse control. (**G**) A model for Retro-EIF2S2 regulation of phenotypic states. Heat shock induced activation of Retro-EIF2S2 can result in either adherent cell phenotypes, if bound to FAU, or if Retro-EIF2S2 is inhibited and unable to bind FAU then a suspension cellular state. (**H**) Schematic summary of Retro-EIF2S2 directed plasticity in HEK293 cells. Adherent vs. suspension cells is contrasted in transfected cells over 120hrs. For B-F triplicate treated cultures are shown with the standard error of the means and p values from a paired T-test.

Retro-EIF2S2 and the truncated Retro-EIF3 exhibit a distinct reconstitution of cell adherence following heat shock (Fig 2E and 2F) but how these transcripts functional drive this phenotype remains unknown. To determine how full-length Retro-EIF2S2 actively modulates cell adherence we generated biotin-labeled variants, transfected cells and performed an immunoprecipation followed by mass-spectrophotometry (IP-MS). The top protein bound to Retro-EIF2S2, following subtraction of the control GFP IP-MS, was the proteasome related proteins 40S ribosomal protein S30 (FAU)(Fig 3A). Interestingly, FAU has been observed previously to modulate anchorage independence in human cells [19]. To validate that indeed FAU is interacting with full-length Retro-EIF2S2, FAU was immunoprecipiated from heat shocked cells and the relative enrichment of full-length Retro-EIF2S2 determined. Full-length Retro-EIF2S2 was found directly bound to FAU following heat shock, indicating that interactions with FAU may underlay the changes in phenotype induced by full-length Retro-EIF2S2 during heat shock (Fig 3B). To determine the requirement of FAU to control cellular phenotypes, cultures were treated with RNA interference to FAU, EIF5A and UBA52, determined previously to bind Retro-EIF2S2 by LC/MS, and the ratio of suspension to adherent cells determined. The reduction of FAU, EIF5A and UBA52 expression (Fig 3C) resulted in a significant reduction in suspension cellular phenotypes (Fig 3D), suggesting that the expression of these proteins is required for HEK293 cells to transition from an adherent to suspension-based phenotype following heat shock.

Both Full-length and the truncated Retro-EIF3 exhibited a distinct reconstitution of cell adherence following heat shock (Fig 2E and 2F) but it remains unknown as to whether these transcripts can function in the absence of heat shock. Over-expression of both the full-length Retro-EIF2S2 and the truncated EIF3 demonstrated an ability to shift the cellular phenotype towards suspension, suggesting that these transcripts can functionally modulate FAU in the absence of heat shock (Fig 3E). Interestingly the truncated EIF3 transcript appeared to more robustly shift cells towards a suspension state relative to full-length Retro-EIF2S2. Similar to full-length Retro-EIF2S2 the truncated EIF3 also interacts with FAU, suggesting that this particular sequence (Fig 2G) is a direct RNA modulator or FAU activity (Fig 3F).

## Discussion

Collectively, the observations presented here suggest that Retro-EIF2S2 modulates adherent cellular phenotypes following heat shock by binding FAU and inhibiting it’s downstream functions, such as proteasomal degradation of proteins (Fig 3F). The loss of Retro-EIF2S2 therefore would result in increased free FAU, and possibly also UBA52 and EIF5A (Fig 3F), which would be expected to lead to, increased proteosomal function, ultimately resulting in a cellular ability to quickly degrade proteins and drive the transition of adherent to suspension cellular phenotypes (Fig 3G). The phenotypic shift observed here with Retro-EIF2S2 and the truncated EIF3 was found to drive the re-adherence of heat shocked HEK293 cells (Fig 3H). Interestingly, the truncated Retro-EIF3, containing the same loop-hairpin structure found in full-length Retro-EIF2S 2 (Fig 2G), binds to FAU, and when over-expressed in cells can also modulate a shift in treated cells from an adherent to suspension state. Notably, the functional region in Retro-EIF2S2 where this structure (EIF3) was derived is in the 3’ end of the gene (S4 Fig) and unique to both the Retro-EIF2S2 locus and it’s protein-coding counterpart endogenous EIF2S2. These observations suggest that Retro-EIF2S2 is a functional non-coding retro-element involved in modulating protein function and that this function may have evolved through particular 3’ motif also found in the EIF2S2 protein-coding transcript. There are several retro-elements/pseudogenes present in the human genome with the many containing only 3’ segments of their protein-coding counterpart, suggesting that this mode of RNA based regulation of protein function may be abundantly present in the cell and may be one of the mechanisms by which natural selection plays out in a regulatory fashion on the genome and evolution of the cell.

## Supporting Information

S1 Fig The effects of heat shock on cell viability. HEK 293 cells were heat shocked (HS) for 30min at 45°C and contrasted with control untreated cells for live cell counts during, immediately after and 24hrs post-heat shock. The averages of triplicate treated cultures are shown with the stand error of the mean and a p value from paired two-sided T-test.
(TIF)

S2 Fig UCSC genome-browser screen shot of the top candidate transcript Retro-Cox6a. A screen shot of the Retro-Cox6a locus is shown along with the local epigenetic marks.
(TIF)

S3 Fig UCSC genome-browser screen shot of the top candidate transcript Retro-Cox6a. A screen shot of the Retro-Cox6a locus is shown (A) uplose and (B) distal.
(TIF)

S4 Fig UCSC genome-browser screen shot of the Retro-EIF2S2 locus. The truncated EIF3 is shown along with those primers used in RIP of FAU for EIF3 binding.
(TIF)

S5 Fig The melt curves for qPCR primers used in assessing the expression of EIF2S2, RetCox6A and RetRPL23a. (TIF)

S1 Table GEO gene analysis from deep sequencing- Genes that were differentially regulated as a result of heatshock are shown. The GEO accession GSE79680.
(XLSX)

S2 Table Primers used in analysis. (*Note lower case nucleotides are not present in the primer but retained in the table to facilitate UCSC BLAT searches).
(PDF)

S3 Table Candidate lncRNAs Retro-EIF2S2, Retro-COX6A, and fragments of Retro-EIF2S2. (PDF)

S4 Table Retro-EIF2S2 and Retro-Cox6a homologous region in protein-coding gene counterparts. The locus of the protein-coding gene counterpart and biological function are shown.
(PDF)

S5 Table Retro_EIF2S2 and Retro_Cox6A homologous predicted pseudogene neighboring or overlapping loci in the human genome. The top candidate matching loci and the neighboring predicted pseudogene is shown for each candidate transcript.
(PDF)
