# Supplementary figures and images for: RNA Directed Modulation of Phenotypic Plasticity in Human Cells
Source: PLoS One. 2016 Apr 15;11(4):e0152424. doi: 10.1371/journal.pone.0152424 (PMC4833343; doi:10.1371/journal.pone.0152424)

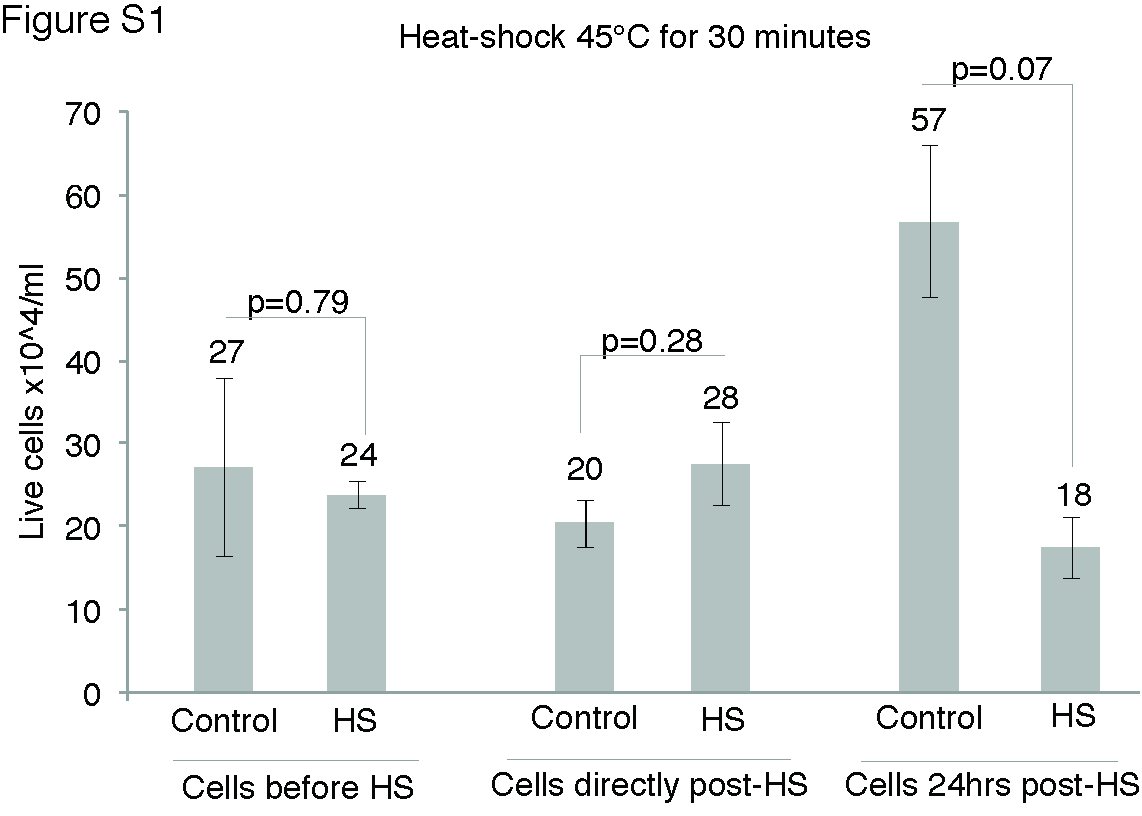

Supplement: S1 Fig — HEK 293 cells were heat shocked (HS) for 30min at 45°C and contrasted with control untreated cells for live cell counts during, immediately after and 24hrs post-heat shock. The averages of triplicate treated cultures are shown with the stand error of the mean and a p value from paired two-sided T-test. (TIF) [file pone.0152424.s001.tif]

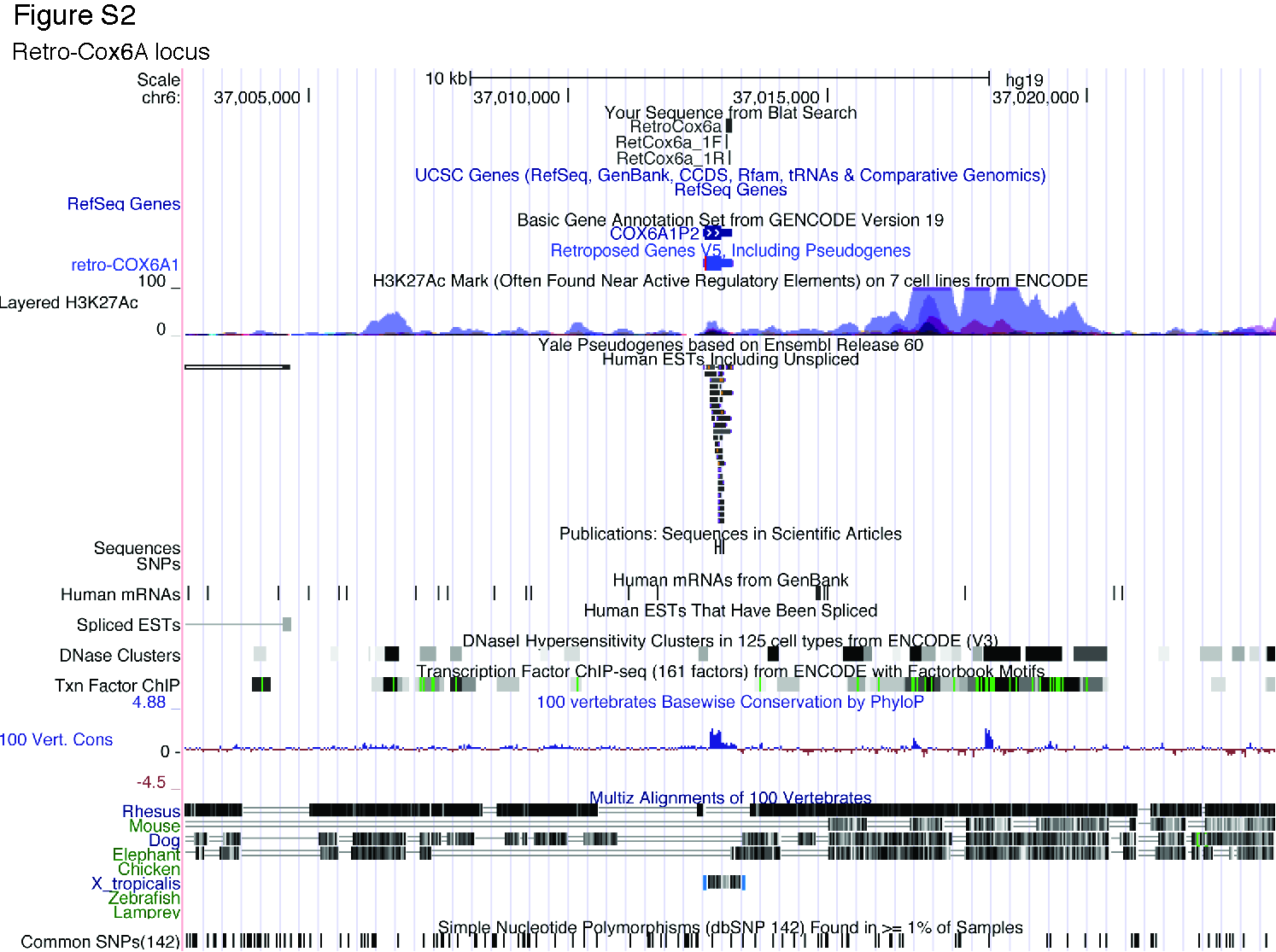

Supplement: S2 Fig — A screen shot of the Retro-Cox6a locus is shown along with the local epigenetic marks. (TIF) [file pone.0152424.s002.tif]

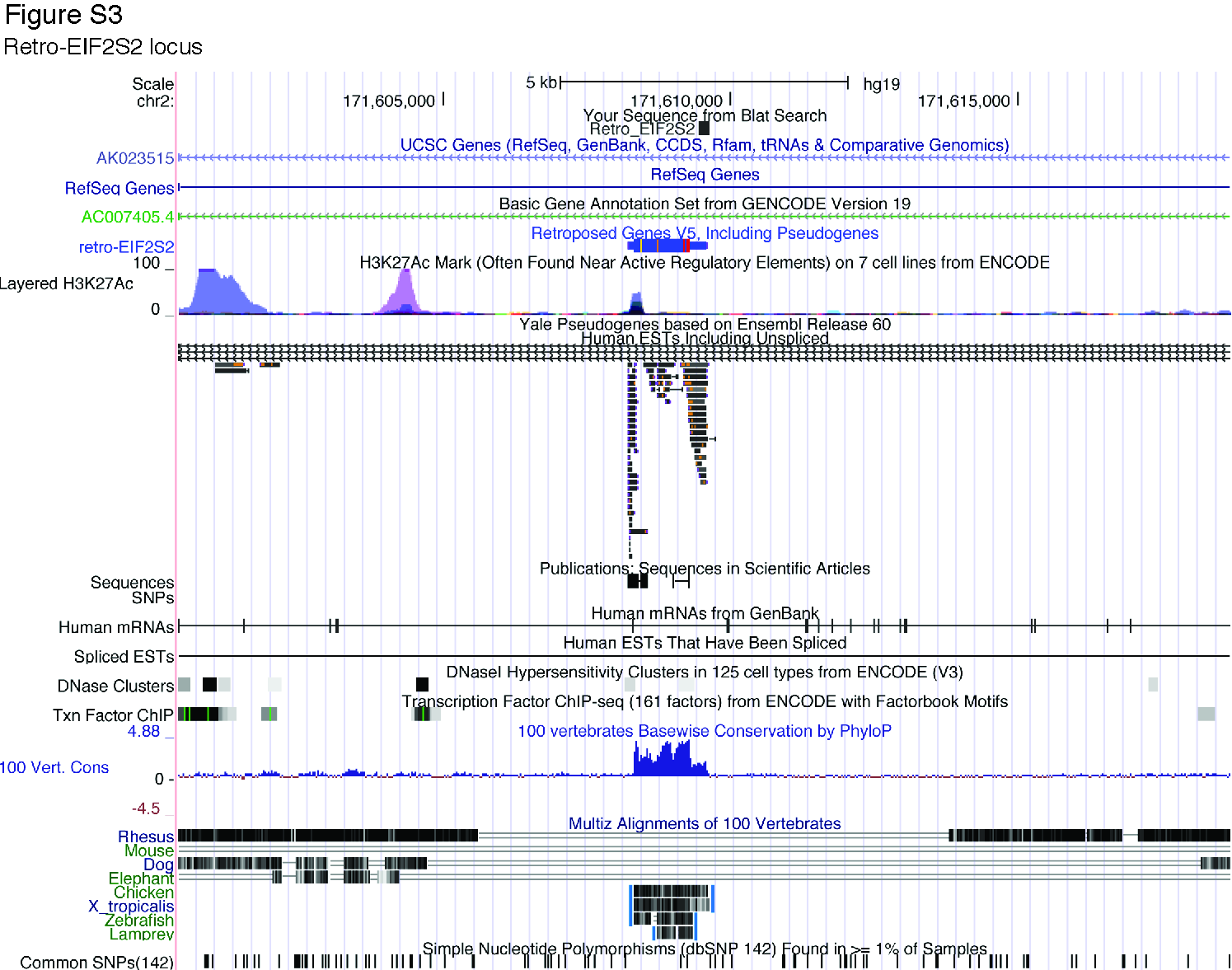

Supplement: S3 Fig — A screen shot of the Retro-Cox6a locus is shown (A) uplose and (B) distal. (TIF) [file pone.0152424.s003.tif]

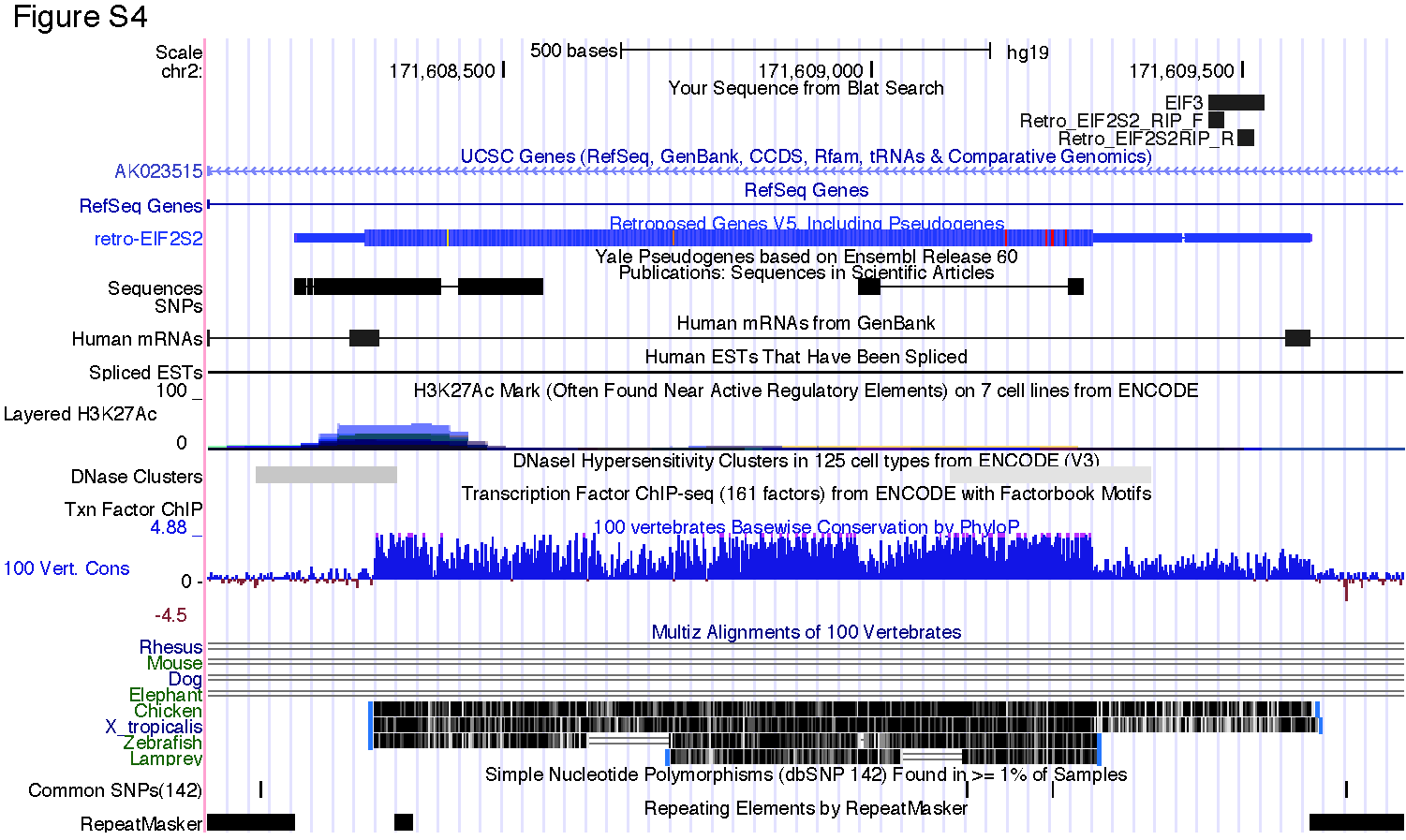

Supplement: S4 Fig — The truncated EIF3 is shown along with those primers used in RIP of FAU for EIF3 binding. (TIF) [file pone.0152424.s004.tif]

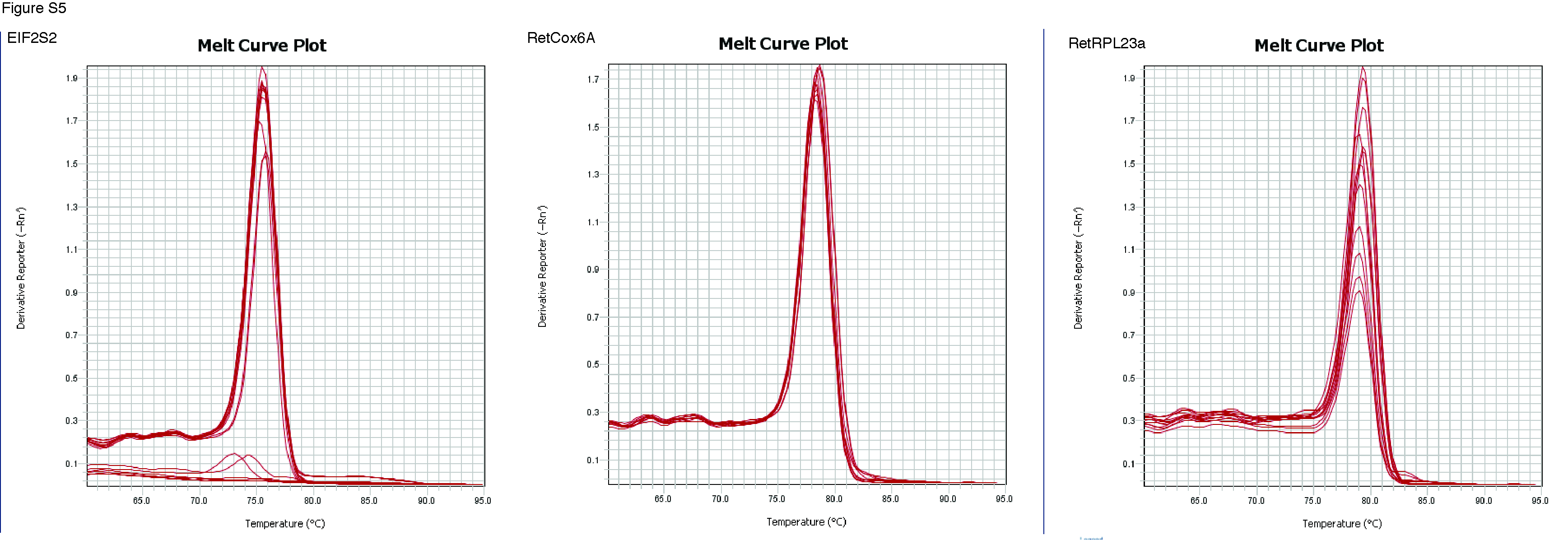

Supplement: S5 Fig — (TIF) [file pone.0152424.s005.tif]
