# Supplementary material for: RNA Directed Modulation of Phenotypic Plasticity in Human Cells
Source: PLoS One. 2016 Apr 15;11(4):e0152424. doi: 10.1371/journal.pone.0152424 (PMC4833343; doi:10.1371/journal.pone.0152424)
Supplement: S2 Table — (*Note lower case nucleotides are not present in the primer but retained in the table to facilitate UCSC BLAT searches). (PDF) [file pone.0152424.s007.pdf]

S2 Table Primers used in analysis. (*Note lower case nucleotides are not present in the primer but retained in the table to facilitate UCSC BLAT searches).

qRT-PCR primers

>SNARE_F1	5'TTTCAACCCATGTGGACCAG3'
>SNARE_R1	5'AACAGGTGGAGCCATTGTGg3'
>SNARE_F2	5'CAGGTTGGCCTCGAACTCgt3'
>SNARE_R2	5'AGCCATTGTGGCTCCAGccg3'
>CA436F1	5'ACAATGGCTCCACCTGTTTt3'
>CA436R1	5'AGGAGCTATTTCTGCTGTTCTT3'
>CA436F2	5'TCCTGGATAAACCTTCCCAAAG3'
>CA436R2	5'TCAGGAAGAAGGGTTTAGTGTTT3'
>RetCox6a_1F	5'GGACCTTAAGCTCACCTTCTTTA3'
>RetCox6a_1R	5'CCATCTCCCGCCACAAGcaaa3'
>RetEIF2S2_F1	5'CCTGTCAGAGAAACGTGATATG3'
>RetEIF2S2_R1	5'AGTTCTAAATACTTCCTAAGTTCCC3'
>RetRPS20_F1	5'TGTTAAGCAGATTACTTCCATCAG3'
>RetRPS20_R1	5'AGTTGACTTAAGCATCTGCAA3'
>RetRPL23a_F1	5'CAGAATTAGGCAGCTGGACTC3'
>RetRPL23a_R1	5'GTTCGACTGGCTCCTGATTAC3'
>FAU_F1	5'GCCAAAGGTGGGCACAAcgt3'
>FAU_R1	5'GGAGAAGAAGAAGAAGAAGACAGG3'
>EIF5A_F1	5'CAGATGACTTGGACTTCGAGAC3'
>EIF5A_R1	5'GACATCTCGACGATCTTACATGG3'
>UBA52_F2	5'CTGTCCTAGATTCTGTCACATCG3'
>UBA52_R2	5'GATCCTCAGTCCCACCAATTAC3'

ChIP assessing retCox6a 3'UTR overlapping region

>retCox6aF2	CACTGGTTTGGACCGTTACT
>retCox6aR2	GAGACCAGTATACCAGTCATCATTT
>retCox6aF3	AATCCACTTCCAACTGGCTAC
>retCox6aR3	TGCAGAGTAACGGTCCAAAC

ChIP assessing RetroCox6a homology containing regions with pseudogene (PGOHUM0000244771)

>retCox6aF4	CATGCTGAATGTGTTCCTGAAG
>retCox6aR4	GCTTGGTCCTGATGCTAAGA
>retCox6aF5	ACTGGCTGCGAAGATGAATAA
>retCox6aR5	GCAAAGCAACGGTCCAAACca
>retCox6aF6	GGCAGGAGGTGGCTTAAATA
>retCox6aR6	AAGCAGGTACGTGACATTCTAA

ChIP assessing RetroCox6a homology containing regions with PGOHUM0000242814

>retCox6aF7	GATGGGACTGAGCTTGCTAAA
>retCox6aR7	GAGATGGGAGTAGGCAATGAAC

>retCox6aF8 CTCTGCACACAGACCAGAAA
>retCox6aR8 GAGATGGAAGATCAGCATAGTAGTC

ChIP assessing EIF2S2 homology containing regions with PGOHUM000023858/AA721424.
>EIF2S2_CHIP_F1 AAAGCACCTCCTTACCTCATATC
>EIF2S2_CHIP_R1 AGTCCTTACAAGACGCTGATG
>EIF2S2_CHIP_F2 GATGCTGGCAACAGAACATCTA
>EIF2S2_CHIP_R2 TTGTCACACGTGCCGATTAC
>EIF2S2_CHIP_F3 CCAGCAACCATACCTGGATT
>EIF2S2_CHIP_R3 CAGGCTCAGAAAGAGACTACAC
(PGOHUM0000248200), on chromosome 14.
>EIF2S2_CHIP_F4 CTGGACAGGTTTGCCATTAGA
>EIF2S2_CHIP_R4 TTCAGACCAACCAACCACTC
>EIF2S2_CHIP_F5 CTCCGTGCCAAAGCTAACTA
>EIF2S2_CHIP_R5 ACCAACCACTCTCCAAACAA
>EIF2S2_CHIP_F6 GTTGGTTGGTCTGAAATCCTTG
>EIF2S2_CHIP_R6 TCCTTATCCCATAGCACGTTTC

For measuring the truncated EIF2S2 RNAs bound to FAU1
>Retro_EIF2S2_TruncF1 TGCTCAAGCTGTTGACATACT
>Retro_EIF2S2_TruncR1 CCCATATCACGTTTCTCTGACA
