# Supplementary material for: RNA Directed Modulation of Phenotypic Plasticity in Human Cells
Source: PLoS One. 2016 Apr 15;11(4):e0152424. doi: 10.1371/journal.pone.0152424 (PMC4833343; doi:10.1371/journal.pone.0152424)
Supplement: S3 Table — (PDF) [file pone.0152424.s008.pdf]

S3 Table Candidate lncRNAs Retro-EIF2S2, Retro-COX6A, and fragments of Retro-EIF2S2.

(1) Retro_COX6A (Full-length_Retro_COX6A) Maps 100% to chr6:37013047-37013174, (also known as COX6A1P2 (pseudogene)

5'TATGGGACCTTAAGCTCACCTTCTTTACTTGTATCAAATGATGACTGGTA TACTGGTCTCCCATCCCTTTGCTTGTGGCGGGAGATGGCTTAAATAAATA ACTTAAACTTAAAAAAATAAAATAAAA3'

(2) Retro_EIF2S2 (full-length transcript) Maps 100% to chr2:171,609,454-171,609,636

5'ATGCTCAAGCTGTTGACATACTCATTGCCTACTTTAACACCTGTCAGAGA AACGTGATATGGGGTAAGGAGGTGCTTTTTTAAAATAGTTCATAGACTTC TGTAAAATGCAAGATAAATTAAAGTTATTATAACAGTGAAAAAAAAGAAA AGGGAACTTAGGAAGTATTTAGAACTAAATGAA3'

Various fragments of Retro_EIF2S2 fragments

(3) Retro_EIF2S2(1)

5'ATGCTCAAGCTGTTGACATACTCATTGCCTACTTTAACACCTGTCAGAGA AACGTGATATGGGGTAAGGAGGTGCTTTTTTAAAATAGTTCATAGACTTC TGTAAAATGCAAGATAAATTAAAGTTATTATAACAGTGAAAAAAAAGAAA3'

(4) Retro_EIF2S2(2)

5'ATGCTCAAGCTGTTGACATACTCATTGCCTACTTTAACACCTGTCAGAGA AACGTGATATGGGGTAAGGAGGTGCTTTTTTAAAATAGTTCATAGACTTC TGTAAAATGCAAGATAAATTA3'

(5) Retro_EIF2S2(3)

5'ATGCTCAAGCTGTTGACATACTCATTGCCTACTTTAACACCTGTCAGAGA AACGTGATATGGGGTAAGGAGGTGCTTT3'

(6) Retro_EIF2S2(4)

5'ATGCTCAAGCTGTTGACATACTCATTGCCTACTTT3'
