# Supplementary material for: RNA Directed Modulation of Phenotypic Plasticity in Human Cells
Source: PLoS One. 2016 Apr 15;11(4):e0152424. doi: 10.1371/journal.pone.0152424 (PMC4833343; doi:10.1371/journal.pone.0152424)
Supplement: S4 Table — The locus of the protein-coding gene counterpart and biological function are shown. (PDF) [file pone.0152424.s009.pdf]

S4 Table Retro-EIF2S2 and Retro-Cox6a homologous region in protein-coding gene counterparts. The locus of the protein-coding gene counterpart and biological function are shown.

**Retrogene**	**Protein-coding locus**	**Function of protein coding gene**	**Locus**
Retro_Cox6a	Cox6A	Cytochrome c oxidase (COX), the terminal enzyme of the mitochondrial respiratory chain, catalyzes the electron transfer from reduced cytochrome c to oxygen	chr12:120,878,423-120,878,529
Retro_EIF2S2	EIF2S2	Eukaryotic translation initiation factor 2 (EIF-2) functions in the early steps of protein synthesis by forming a ternary complex with GTP and initiator tRNA and binding to a 40S ribosomal subunit.	chr6:121,102,194-121,103,413
