# Supplementary material for: RNA Directed Modulation of Phenotypic Plasticity in Human Cells
Source: PLoS One. 2016 Apr 15;11(4):e0152424. doi: 10.1371/journal.pone.0152424 (PMC4833343; doi:10.1371/journal.pone.0152424)
Supplement: S5 Table — The top candidate matching loci and the neighboring predicted pseudogene is shown for each candidate transcript. (PDF) [file pone.0152424.s010.pdf]

S5 Table Retro_EIF2S2 and Retro_Cox6A homologous predicted pseudogene neighbouring or overlapping loci in the human genome. The top candidate matching loci and the neighbouring predicted pseudogene is shown for each candidate transcript.

**Retrogene**	**Overlapping pseudogene**	**Locus**
Retro_Cox6a	Retro_Cox6A seed locus	chr6:37,013,048-37,013,174
	PGOHUM0000243758	chr6:121,102,194-121,103,413
	PGOHUM0000244771	chr1:80,961,502-80,962,621
	PGOHUM0000242814	chr11:80,668,338-80,668,951
	PGOHUM0000233548	chr7:123,572,319-123,572,968
Retro_EIF2S2	Retro_EIF2S2 seed locus	chr2:171609454-171609636
	PGOHUM0000238585	chr10:94,428,105-94,428,945
	PGOHUM0000248200	chr10:94,428,105-94,428,945
	PGOHUM0000237895	chr3:184,962,830-184,963,725
	PGOHUM0000242822	chr11:82,548,068-82,548,578
